# REGIA, An EU Project on Functional Genomics of Transcription Factors From *Arabidopsis Thaliana*

**DOI:** 10.1002/cfg.146

**Published:** 2002-04

**Authors:** Javier Paz-Ares, The REGIA Consortium

**Affiliations:** Centro Nacional de Biotecnología-CSIC. Campus de Cantoblanco Madrid 28049 Spain

## Abstract

Transcription factors (TFs) are regulatory proteins that have played a pivotal role in the evolution of eukaryotes and that also have great biotechnological potential. REGIA (REgulatory Gene Initiative in Arabidopsis) is an EU-funded project involving 29 European laboratories with the objective of determining the function of virtually all transcription factors from the model plant, *Arabidopsis thaliana*. REGIA involves: 1. the definition of *TF* gene expression patterns in *Arabidopsis*; 2. the identification of mutations at *TF* loci; 3. the ectopic expression of TFs (or derivatives) in *Arabidopsis* and in crop plants; 4. phenotypic analysis of the mutants and mis-expression lines, including both RNA and metabolic profiling; 5. the systematic analysis of interactions between TFs; and 6. the generation of a bioinformatics infrastructure to access and integrate all this information. We expect that this programme will establish the full biotechnological potential of plant TFs, and provide insights into hierarchies, redundancies, and interdependencies, and their evolution. The project involves the preparation of both a *TF* gene array for expression analysis and a normalised full length open reading frame (ORF) library of TFs in a yeast two hybrid vector; the applications of these resources should extend beyond the scope of this programme.


**Comparative and Functional Genomics**
*Comp Funct Genom* 2002; **3**: 102–108.
Published online 1 March 2002 in Wiley InterScience (www.interscience.wiley.com). **DOI:** 10.1002/cfg.146

**Conference Review**

### REGIA, an EU project on functional genomics of transcription factors from *Arabidopsis thaliana*

Javier Paz-Ares* and The REGIA Consortium†
*Centro Nacional de Biotecnología-CSIC. Campus de Cantoblanco, 28049 Madrid, Spain*

*Correspondence to: Centro Nacional de Biotecnología-CSIC. Campus de Cantoblanco, 28049 Madrid, Spain.
E-mail: jpazares@cnb.uam.es

†A full list of authors and addresses appears at the end of this paper.

Received: 24 January 2002
Accepted: 29 January 2002

### Abstract

**Transcription factors (TFs) are regulatory proteins that have played a pivotal role in the evolution of eukaryotes and that also have great biotechnological potential. REGIA (REgulatory Gene Initiative in Arabidopsis) is an EU-funded project involving 29 European laboratories with the objective of determining the function of virtually all transcription factors from the model plant, *Arabidopsis thaliana*. REGIA involves: 1. the definition of *TF* gene expression patterns in *Arabidopsis*; 2. the identification of mutations at *TF* loci; 3. the ectopic expression of TFs (or derivatives) in *Arabidopsis* and in crop plants; 4. phenotypic analysis of the mutants and mis-expression lines, including both RNA and metabolic profiling; 5. the systematic analysis of interactions between TFs; and 6. the generation of a bioinformatics infrastructure to access and integrate all this information. We expect that this programme will establish the full biotechnological potential of plant TFs, and provide insights into hierarchies, redundancies, and interdependencies, and their evolution. The project involves the preparation of both a *TF* gene array for expression analysis and a normalised full length open reading frame (ORF) library of TFs in a yeast two hybrid vector; the applications of these resources should extend beyond the scope of this programme.** **Copyright © 2002 John Wiley & Sons, Ltd.**

**Keywords:** REGIA; Arabidopsis; transcription factors; functional genomics; array; two-hybrid system; insertion mutant

### Introduction

The completion of the sequence of the genome of *Arabidopsis* [28] represents one of the most significant landmarks in the history of plant biology. The next step, the interpretation of this information in functional terms is a demanding task in terms of resources. One obvious class of proteins to be of top priority are transcription factors.

These regulatory proteins provide the most common mechanism of regulation of co-ordinated gene activity, transcriptional control. Because of their power to control gene expression and consequently complex traits, TFs are believed to have played an important role in the evolution of plants and have been the targets for breeding and domestication [reviewed in 3]. Thus, many of the best characterised QTLs (quantitative trait loci) and agronomically important genes correspond to TFs (for example, [2,15,16,20,21,29,31]). Two examples of the significance of TFs in domestication and breeding are *TEOSINTE BRANCHED* and the *GRAS* genes. *TEOSINTE BRANCHED1* is a bHLH type TF, responsible, through its regulatory activity, for most of the morphological difference between maize and its wild ancestor, teosinte [2]. The *GRAS* genes are responsible for the reduced size and increased grain yield of cereals bred for the 'green revolution' and encode mutant giberellin response modulators which are thought to act as TFs [20,21]. Mutations in some genes encoding TFs can phenocopy inter- or intra-specific natural variants [9,1] emphasising their importance in evolution as well.

As a corollary of their seminal role in the determination of plant traits, transcription factors are

Copyright © 2002 John Wiley & Sons, Ltd.

considered to have enormous biotechnological potential for the manipulation of agronomic traits. In support of this are the established examples in which transcription factors have been used to manipulate plant metabolism (for example, the anthocyanin, phlobaphene and lignin pathways; [14,5,27]), development (for example, flowering time and cell shape; [4,17,19,30]) and responses to stress (for example, cold, salinity, and drought stresses; [6,13]).

TFs are endowed with characteristics that make them particularly suitable for driving evolution and for biotechnological exploitation. One of these characteristics is modularity, whereby domains within the proteins function independently, thus facilitating module exchange between TFs [10] and allowing for the engineering of new TF activities. Another characteristic of TFs is the large degree of functional redundancy in higher eukaryotes, including plants. Transcription factors are, in general, members of large families that often include closely related genes that are also functionally related. Within subfamilies different extents of partial redundancy are to be expected, in which redundant genes may diverge in their expression pattern, generally due to mutations in their cis-regulatory regions [10]. Extreme examples of redundancy and of divergence are the cases of *SEP1*, *SEP2* and *SEP3*, and of *GL1* and *WER*, respectively [12,18]. In the first case, only the triple mutant *sep1*, *sep2*, *sep3* showed a phenotypic difference with the wild type (sepaloid flowers). In the second case, mutations at *GL1* affect trichome formation, whereas *wer* mutations affect root hair formation, but their proteins are functionally equivalent. Finally, these two characteristics, modularity and redundancy, together with the fact that transcription factors tend to act downstream in signal transduction pathways limits pleiotropy of mutations in transcription factors, a necessary condition for evolutionary and biotechnological potential [3].

### Structure of the REGIA project

Current functional studies are generally based on the identification of mutations at the loci of interest and the evaluation of the phenotypic effects of these mutations. This primary strategy is often complemented by the generation of transgenic plants ectopically expressing the corresponding gene (or a derivative) and their phenotypic characterisation. Our approach rests on similar principles, but has been adapted and extended to cope with and to exploit the characteristics common to many transcription factors, such as low abundance, (partial) redundancy, functional interdependency and modularity. The fact that TFs regulate gene expression, and that expression is particularly amenable to molecular analysis, means that we have also been able to include aspects of target gene identification in our functional analyses. Briefly, the activities (or workpackages) in the REGIA project are the following:

- The analysis of the *Arabidopsis* genome sequence, the analysis of all genes encoding recognisable TFs and phylogenetic analysis, and the isolation of unique identifier probes to prepare a TF gene array and the analysis of the expression patterns of TF genes. (WP1)
- The identification of mutants of a large number of strategically identified TF genes through reverse genetic screens (WP2)
- The ectopic expression of selected TF genes (or derivatives, including inducible versions) in *Arabidopsis* and key crop species (WP3)
- Phenotypic analysis, including RNA and metabolic profiling, of plants mutated at TF loci or ectopically expressing TF genes (or their derivatives) to define their biological functions (WP4)
- The systematic analysis of physical (protein–protein) interactions between TFs (WP5)
- The bioinformatic analysis and management of data produced in the programme (WP6)
- The management and co-ordination of the scientific activities on the programme, their communication to other scientific groups and industry and the protection of IP generated during the programme. (WP7)

The relationships between the objectives of the project and the workpackages, as diagrammatically shown in Figure 1, are:

- Information on the function of TFs will come primarily from the phenotypic analysis (WP4) of *Arabidopsis* plants with mutations at TF loci (WP2) or lines ectopically expressing TFs (or their derivatives; WP3). Expression data on TFs (WP1) will assist phenotypic analysis. The bioinformatic exercises of genome mining and phylogenetic analyses have helped to simplify TF gene families into functionally related subfamilies. Functional characterisation of any one member

Copyright © 2002 John Wiley & Sons, Ltd.

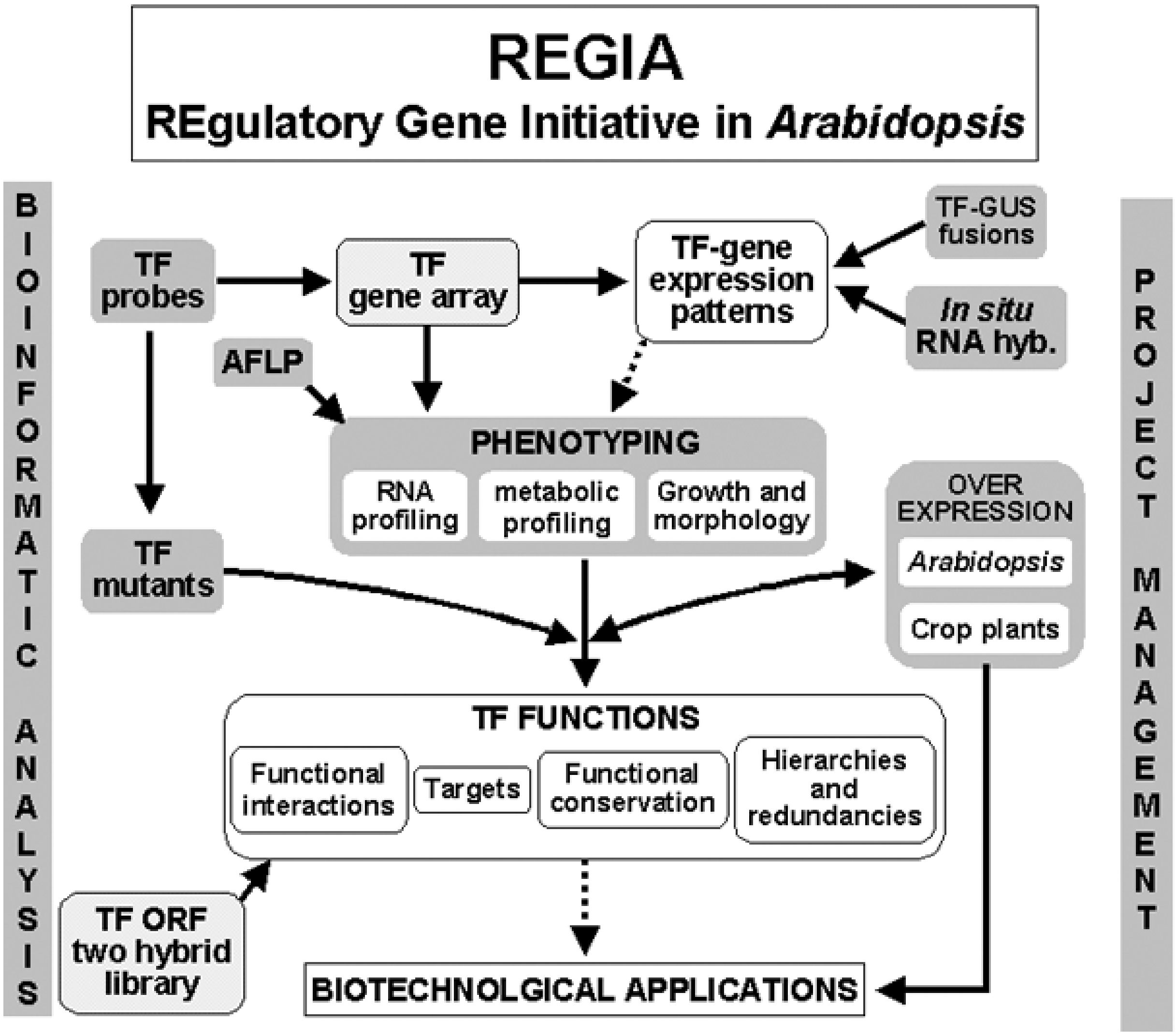

**Figure 1.** Relationships between the activities in the REGIA project. The white boxes and dashed arrows reflect informational outputs of the programme on expression patterns, functions and biotechnological applications. The dark grey boxes reflect the activities towards obtaining this information and the light grey outlined boxes reflect the two resources that will be produced by the programme of such subfamilies, coupled with expression analysis of all subfamily members should permit preliminary functional assignment to the entire subfamily membership.

- Information on regulatory hierarchies will come primarily from the analysis of the effects of mutants/overexpressors of a given TF on the expression of other TFs (WP4).
- Insights into redundancies among TFs will be obtained from the analysis of RNA profiles of TF mutants (WP4). If mutations in any of two structurally related TF genes, or their hyper-expression, influence the expression of a given (target) gene, these TFs are potentially redundant. Hints on redundancy will also come from detection of overlapping expression patterns of closely related TFs (WP1). Confirmation of redundancy will be obtained by preparing and analysing the corresponding double mutants.
- Insights into functional conservation and potential agronomic uses will be obtained from comparison of the effects of ectopic expression

Copyright © 2002 John Wiley & Sons, Ltd.

of a given TF (or a derivative) in *Arabidopsis* and in other species (tomato, rapeseed, maize and soybean; WP3&4).

- Information on functional interdependencies will be obtained primarily from the studies of the interactions between TFs, using the yeast two-hybrid system. Hints on interdependences (i.e, on indirect as well as direct interactions) will also come from the studies on functional links between TFs based on molecular phenotypes of the respective TF mutants (WP4). Thus, if mutations at two or more TF loci, (or their hyper-expression), influence the expression of the same target gene, these TFs are possibly functionally interdependent. The exhaustive definition of interdependencies following this second criterion is beyond the scope of this proposal, as it would require the detailed functional characterisation of each of the more than 1500 TF genes present in *Arabidopsis*. ( [22], REGIA, unpublished). Confirmation of interdependencies between two TFs identified by the yeast two-hybrid screen will be obtained through the analysis of transgenic plants hyperexpressing the two TFs.

Obviously, the biotechnological potential of each TF will depend very much on the trait it controls (WP4), but clearly the determination of functional interdependencies forms a foundation for the exploitation of the biotechnological potential of transcription factors. In fact, it is the functional and biotechnological relevance of TF interdependencies that, in our opinion, justifies the huge amount of work involved in their study, and consequently the large size and the European dimension to the Consortium.

### Organisation

The Consortium includes one Project Manager and 29 research groups, 27 from academia and 2 from industry. One of the group leaders is the scientific coordinator and another seven group leaders are workpackage coordinators, forming, together with the Project Manager, the Coordination Committee. The activities are organised around several core centres which provide support to all groups on techniques which could not be efficiently implemented at the level of individual laboratories (for example, the preparation of TF cDNA and EST arrays, *in situ* RNA hybridisation analysis of TF gene expression, high throughput AFLP-based transcript profiling, metabolic profiling of mutant and/or transgenic plants, generation of transgenic crop plants, high throughput two-hybrid based study of TF interactions and bioinformatic analysis (see Alonso-Allende *et al.*, this issue). Some of the activities being undertaken in individual laboratories transcend the group's specific interests in particular types of transcription factor (for instance, they provide probes and full size ORFs cloned in two-hybrid vectors for a subset of TF genes). The group benefits from this additional investment because the tools they are developing provide a better understanding of the function of specific TFs in the context of transcriptional control in plants as a whole. In this way, there will be an efficient use of resources, which will also benefit the individual research interests of the different laboratories, reinforcing their intellectual freedom and creativity.

### Progress

The program is halfway through at present and much of the data that has been obtained is still fragmentary, but it already provides indications on its potential to uncover TF gene function and application.

A significant result of our studies has been the thorough bioinformatic definition and phylogenetic analysis of some transcription factor families, which provide a basis for identifying redundancy and for functional assignment when information on function from highly related genes from other species or from the same species is available (see for instance, [7,26]).

One notable aspect of our approach is the exhaustive phenotypic analysis of TF mutants which, in addition to standard phenotypic screenings, includes RNA profiling, using DNA arrays complemented by AFLP-based techniques, and metabolic profiling. These techniques are currently operative in the Consortium. This strategy should allow functional links between TFs to be established, as well as links between 'genes and metabolites', using bioinformatics tools. In addition, such powerful phenotypic analysis should help to solve analytical problems with gene redundancy since we will be able to detect even the minor phenotypic effects which can arise when there is a (partially) redundant counterpart of the mutant TF under

Copyright © 2002 John Wiley & Sons, Ltd. 

study. An example of this is AtMYB4, for which no phenotype was obvious in mutant plants grown under standard conditions. Expression profiling showed it to change most in expression in plants exposed to UV-B light. When mutants were grown under UV-B light they were more tolerant than wild type plants to this stress, establishing a role for this TF in the negative regulation of UV-B protection [11,8]. We have also prepared a TF gene array that will allow the determination of TF expression patterns. This will provide clues to functions (for example, TFs controlling the cell cycle) and assist phenotypic analysis of mutants. In addition, it will allow the detection of overlapping expression patterns among related and potentially redundant TFs, thereby providing a rational basis for the selection of the double mutants to be prepared and analysed.

The fact that TFs are usually functionally interdependent and act in combinations rather than alone [25], has been given special consideration in the context of this programme. The importance of characterising TF functional interdependency is twofold: first, it will help to define regulatory networks, and second, it is a necessary step to permit the full manifestation of TF regulatory (and biotechnological) potential. For instance, the maize C1 and R anthocyanin regulatory genes are known to interact and it has been shown that their co-expression in transgenic *Arabidopsis* is necessary for anthocyanin production in all tissues [14]. Functional interdependencies involve both direct and indirect interactions. Our approach to study direct interactions depends on a novel iterative screening of interactions among TFs based on the use of the yeast two-hybrid system, and we are generating a normalised full size TF library (800 full length ORFs cloned at present) which will be made available to the scientific community for screening for additional interactions, particularly with non-TF proteins. Additional clues on direct and, especially, on indirect interactions will come from the studies on functional links based on the molecular phenotypes of TF mutants.

Also important in the context of this proposal is the modular organisation of TFs, whereby the selector, DNA-binding domain, and the effector (activation or repression) domain are to a great extent functionally independent, allowing module exchange and/or the addition of other modules (for instance, conferring chemical control, as demonstrated in several instances including the *Arabidopsis AP3* gene, controlling petal and stamen formation, *CONSTANS* gene, controlling flowering time, and the *Arabidopsis STM* gene, controlling meristem identity; [24,23], Sablowski, personal communication). We have taken advantage of TF modularity to prepare TFs whose activity can be post-translationally controlled with glucocorticoids, or whose effector domain is replaced by a strong constitutive activation or repression domain. In this way, we expect to circumvent possible problems of redundancy, or those derived from expression of constitutively active transcription factors (such as lethality), as well as of problems associated with factors for which activity depends on an unknown post-translational modification/interaction. Additionally, inducible constructs such as those activated post-translationally by supply of steroids will allow for the identification of direct target genes of particular TFs. Identification of target genes of particular TFs will be possible using the mutant and inducible lines and microarray analysis or cDNA-AFLP. These activities will help characterise function further. Once regulatory frameworks have been defined for *Arabidopsis*, it is anticipated that we will be able to modify the activity of relevant TFs in crop plants to engineer desirable traits.

In summary, we have already defined many transcription factor families through bioinformatic analysis, the TF gene array has already been prepared and is being used by the different groups for expression analysis under 72 defined conditions/treatments. More than 150 transcription factor mutants have been isolated, and more than 250 TF-derived constructs have been prepared and introduced into transgenic plants which are currently being analysed. Metabolic profiling techniques have been set up. In addition, more than 800 full length TF ORFs have been cloned in Gateway entry vectors and transferred to two-hybrid delivery vectors. It is expected that by the end of the program these activities will have crystallised to provide many new ideas on plant transcription factor function. It is also important that a very significant degree of integration of the activities of 30 European laboratories has been achieved in a relatively short time. These integrated activities have been undertaken by groups separated by large distances and by many who have not worked in such co-ordinated programmes previously. We expect the full manifestation of the potential of this Consortium to be evident within the next three years.

Copyright © 2002 John Wiley & Sons, Ltd.

### The REGIA Consortium

### Names

Alfonso Valencia[1] Paolo Costantino[2], Paola Vittorioso[2] Brendan Davies[3] Phil Gilmartin[3] Jerome Giraudat[4] François Parcy[4] Andreas Reindl[5] Robert Sablowski[6] George Coupland[6a] Cathie Martin[6] Gerco C. Angenent[7] Helmut Baumlein[8], Hans-Peter Mock[8] Pilar Carbonero[9] Lucia Colombo[10], Chiara Tonelli[10] Peter Engström[11] Wolfgang Droege-Laser[12], Christiane Gatz[12] Tony Kavanagh[13] Sergei Kushnir[14], Marc Zabeau[14] Thomas Laux[15] Mike Hordsworth[16] Ida Ruberti[17] Frank Ratcliff[18] Sjef Smeekens[19] Imre Somssich[20], Bernd Weisshaar[20] Jan Traas[21]

### Addresses

[1]Centro Nacional de Biotecnología-CSIC. Campus de Cantoblanco, 28049 Madrid, Spain. [2]Dip. Genetica e Biologia Moleculare, Universita di Roma 'La Sapienza', P. le A. Moro 5, 00185 Rome, Italy. [3]Leeds Institute for Plant Biotechnology and Agriculture, Centre for Plant Sciences, University of Leeds, Leeds LS2 9JT, UK. [4]Institut des Sciences Vegetales, CNRS, Batiment 23, Av. De la Terrasse, 91198 Gir-sur-Yvette CEDEX, France. [5]BASF AG, Plant Biotechnology and Crop Protection, ZHP/T, A30, 67057 Ludwigshagen, Germany. [6]Department of Molecular Genetics, John Innes Centre, Norwich Research Park, Colney, Norwich, NR4 7UH, UK. [7]CPRO-DLO, P.O. Box 16, 6700 AA Wageningen, The Netherlands. [8]Institut für Pflanzengenetik und Kulturpflanzenforschung, Corrensstrasse 3, D-06466 Gatersleben, Germany. [9]Dept. Bioquímica y Biología Molecular, ETSI Agrónomos, Universidad Politécnica, Cuidad Universitaria, 28040 Madrid, Spain. [10]Dipartimento di Genetica e di Biologia dei microrganismi, Università degli Studi di Milano, Via Celoria 26, 20133 Milano, Italy. [11]Department of Physiological Botany, Uppsala University, Villavagen 6, S-75236 Uppsala, Sweden. [12]Albrecht-von-Haller-Institut für Pflanzenwissenschaften, Abteilung für Allgemeine und Enwicklungsphysiologie AG Gatz, Universität Göttingen, Untere Karspuele 2, D-37073 Göttingen, Germany. [13]Department of Genetics, Trinity College, Dublin 2, Ireland. [14]Vlaams Instituut voor Biotechnology (VIB), Department of Genetics, University of Gent, K.L. Ledeganckstraat 35, 9000 Gent, Belgium [15]Institut fuer Biologie III, Universitaet Freiburg, Schaenzlestr. 1, 79104 Freiburg, Germany [16]Institute of Arable Crops Research, Long Ashton Research Station, University of Bristol, Department of Agricultural Sciences, Long Ashton, Bristol BS41 9AF, UK. [17]Centro di studio per gli Acidi Nucleici, c/o Dipartimento di Genetica e Biologia Moleculare, Universita di Roma 'La Sapienza', P. le Aldo Moro 5, 00185 Rome, Italy. [18]Zeneca Agrochemicals, John Innes Centre, Norwich Research Park, Colney, Norwich NR4 7UH, UK. [19]Molecular Plant Physiology, University of Utrecht, Padualaan 8, 3584 Utrecht, The Netherlands. [20]Max-Planck-Institut fur Zuchtungsforschung, Carl-von-Linne-Weg 10, D-50829 Koln, Germany. [21]INRA, Laboratoire de Biologie Cellulaire, Route de St-Cyr, 78026 Versailles, France.

[a]Present address: Max-Planck-Institut fur Zuchtungsforschung, Carl-von-Linne-Weg 10, D-50829 Koln, Germany.

### Acknowledgement

The REGIA project is funded by the EU (contract number QLG-CT11999-00876)

### References

1. Cubas P, Vincent C, Coen E. 1999. An epigenetic mutation responsible for natural variation in floral symmetry. *Nature* **401**: 157–161.
2. Doebley J, Stec A, Hubbard L. 1997. The evolution of apical dominance in maize. *Nature* **386**: 485–488.
3. Doebley J, Lukens L. 1998. Transcriptional regulators and the evolution of plant form. *Plant Cell* **10**: 1075–1082.
4. Glover BJ, Pérez-Rodriquez M, Martín C. 1998. Development of several epidermal cell types can be specified by the same MYB-related plant transcription factor. *Development* **125**: 3497–3508.
5. Grotewold E, Chamberlin M, Snook M, *et al.* 1998. Engineering secondary metabolism in maize cells by ectopic expression of transcription factors. *Plant Cell* **10**: 721–740.
6. Jaglo-Ottosen KR, Gilmour SJ, Zarka DG, Schabenberger O, Thomashow MF. 1998. *Arabidopsis* CBF1 overexpression induces COR genes and enhances freezing tolerance. *Science* **280**: 104–106.
7. Jakoby M, Vicente-Carbajosa J, Dröge-Laser W, *et al.* The family of bZIP transcription factors in *Arabidopsis thaliana. Trends in Plant Science* **0**: 00–00. [VOLUME AND PAGE NUMBERS NEEDED]
8. Jin H, Cominelli E, Bailey P, *et al.* 2000. Transcriptional repression by AtMYB4 controls production of UV- protecting sunscreens in *Arabidopsis*. *EMBO J* **19**: 6150–6161.
9. Kempin SA, Savidge B, Yanofsky MF. 1995. Molecular basis of the *cauliflower* phenotype in *Arabidopsis*. *Science* **267**: 522–525.

Copyright © 2002 John Wiley & Sons, Ltd.

10. Kirschner M, Gerhart J. 1998. Evolvability. *Proc Natl Acad Sci U S A* **95**: 8420–8427.
11. Kranz HD, Denekamp M, Greco R, *et al*. 1998. Towards functional characterisation of the members of the *R2R3-MYB* gene family from *Arabidopsis thaliana*. *Plant J* **16**: 263–276.
12. Lee MM, Schiefelbein J. 2001. Developmentally distinct MYB genes encode functionally equivalent proteins in *Arabidopsis*. *Development* **128**: 1539–1546.
13. Liu Q, Kasuga M, Sakuma Y, *et al*. 1998. Two transcription factors, DREB1 and DREB2, with an EREBP/AP2 DNA binding domain separate two cellular signal transduction pathways in drought- and low-temperature-responsive gene expression, respectively, in *Arabidopsis*. *Plant Cell* **10**: 1391–1406.
14. Lloyd AM, Walbot V, Davis RW. 1992. *Arabidopsis* and *Nicotiana* anthocyanin production activated by maize regulators R and C1. *Science* **258**: 1773–1775.
15. McMullen MD, Byrne PF, Snook ME, *et al*. 1998. Quantitative trait loci and metabolic pathways. *Proc Natl Acad Sci U S A* **95**: 1996–2000.
16. Michaels SD, Amasino RM. 1999. FLOWERING LOCUS C encodes a novel MADS domain protein that acts as a repressor of flowering. *Plant Cell***11**: 949–956.
17. Payne T, Clement J, Arnold D, Lloyd A. 1999. Heterologous myb genes distinct from *GL1* enhance trichome production when overexpressed in *Nicotiana tabacum*. *Development* **126**: 671–682.
18. Pelaz S, Ditta GS, Baumann E, Wisman E, Yanofsky MF. 2000. B and C floral organ identity functions require SEPALLATA MADS-box genes. *Nature* **405**: 200–203.
19. Pena L, Martin-Trillo M, Juarez J, *et al*. 2001. Constitutive expression of *Arabidopsis* LEAFY or APETALA1 genes in citrus reduces their generation time. *Nat Biotechnol* **19**: 263–267.
20. Peng J, Carol P, Richards DE, *et al*. 1997. The *Arabidopsis GAI* gene defines a signaling pathway that negatively regulates gibberellin responses. *Genes Dev* **11**: 3194–3205.
21. Peng J, Richards DE, Hartley NM, *et al*. 1999. Green revolution genes encode mutant gibberellin response modulators. *Nature* **400**: 256–261.
22. Riechmann JL, Heard J, Martin G, *et al*. 2000. *Arabidopsis* transcription factors: genome-wide comparative analysis among eukaryotes. *Science* **290**: 2105–2110.
23. Sablowski RW, Meyerowitz EM. 1998. A homolog of *NO APICAL MERISTEM* is an immediate target of the floral homeotic genes *APETALA3/PISTILLATA*. *Cell* **92**: 93–103.
24. Simon R, Igeno MI, Coupland G. 1996. Activation of floral meristem identity genes in *Arabidopsis*. *Nature* **384**: 59–62.
25. Singh KB. 1998. Transcriptional regulation in plants: the importance of combinatorial control. *Plant Physiol* **118**: 1111–1120.
26. Stracke R, Werber M, Weisshaar B. 2001. The R2R3-MYB gene family in *Arabidopsis thaliana*. *Curr Opin Plant Biol* **4**: 447–456.
27. Tomagnone L, Merida A, Parr A, *et al*. 1998. The AmMYB308 and AmMYB330 transcription factors from antirrhinum regulate phenylpropanoid and lignin biosynthesis in transgenic tobacco. *Plant Cell* **10**: 135–154.
28. The Arabidopsis Genome Initiative. 2000. Analysis of the genome sequence of the flowering plant *Arabidopsis thaliana*. *Nature* **14**: 796–815.
29. Thornsberry JM, Goodman MM, Doebley J, *et al*. 2001. Dwarf8 polymorphisms associate with variation in flowering time. *Nat Genet* **28**: 286–289.
30. Weigel D, Nilsson O. 1995. A developmental switch sufficient for flower initiation in diverse plants. *Nature* **377**: 495–500.
31. Yano M, Katayose Y, Ashikari M, *et al*. 2000. Hd1, a major photoperiod sensitivity quantitative trait locus in rice, is closely related to the *Arabidopsis* flowering time gene CONSTANS. *Plant Cell* **12**: 2473–2484.

Copyright © 2002 John Wiley & Sons, Ltd. 
